# Eu^3+^ and Tb^3+^ @ PSQ: Dual Luminescent Polyhedral Oligomeric Polysilsesquioxanes

**DOI:** 10.3390/ma15227996

**Published:** 2022-11-12

**Authors:** Stefano Marchesi, Ivana Miletto, Chiara Bisio, Enrica Gianotti, Leonardo Marchese, Fabio Carniato

**Affiliations:** 1 Dipartimento di Scienze e Innovazione Tecnologica, Università del Piemonte Orientale, Viale Teresa Michel, 11, 15121 Alessandria, Italy; 2 Dipartimento di Scienze del Farmaco, Università del Piemonte Orientale, Largo Donegani, 2/3, 28100 Novara, Italy; 3 CNR-SCITEC Istituto di Scienze e Tecnologie Chimiche “Giulio Natta”, Via G. Venezian, 21, 20133 Milano, Italy; 4 Dipartimento per lo Sviluppo Sostenibile e la Transizione Ecologica, Università del Piemonte Orientale, Piazza Sant’Eusebio, 5, 13100 Vercelli, Italy

**Keywords:** silsesquioxane, polysilsesquioxane, POSS, lanthanide, europium, terbium, coordination, luminescence, energy transfer, co-doped material

## Abstract

The synthesis and characterization of novel luminescent amorphous POSS-based polysilsesquioxanes (PSQs) with Tb^3+^ and Eu^3+^ ions directly integrated in the polysilsesquioxane matrix is presented. Two different Tb^3+^/Eu^3+^ molar ratios were applied, with the aim of disclosing the relationships between the nature and loading of the ions and the luminescence properties. Particular attention was given to the investigation of site geometry and hydration state of the metal centers in the inorganic framework, and of the effect of the Tb^3+^ → Eu^3+^ energy transfer on the overall optical properties of the co-doped materials. The obtained materials were characterized by high photostability and colors of the emitted light ranging from orange to deep red, as a function of both the Tb^3+^/Eu^3+^ molar ratio and the chosen excitation wavelength. A good energy transfer was observed, with higher efficiency displayed when donor/sensitizer concentration was lower than the acceptor/activator concentration. The easiness of preparation and the possibility to finely tune the photoluminescence properties make these materials valid candidates for several applications, including bioimaging, sensors, ratiometric luminescence-based thermometers, and optical components in inorganic or hybrid light-emitting devices.

## 1. Introduction

Polyhedral oligomeric silsesquioxanes (or POSS) represent a unique class of three-dimensional organo-silicon compounds with a generic molecular structure consisting of an inorganic cage having different degrees of symmetry and hydrogen atoms or organic pendant arms bound to the apexes of the core framework. The term “silsesquioxane” derives from their intimate chemical nature, in which silicon atoms are covalently bounded to one-half oxygen (sesqui-) and hydrocarbon (or hydrogen) units (-ane), thus generating a 3D siloxane (Si-O-Si) skeleton of tetrahedral units with a base chemical formula (RSiO_3/2_)*_n_* (*n* = 4–12; R = H or organic substituents) [1,2,3].

The most common cage-shaped silsesquioxanes are the completely condensed POSS with a typical cubic R_8_Si_8_O_12_ structure [1,2], while incompletely condensed POSS bearing in the structure reactive silanols (Si-OH) (i.e., trisilanol R_7_Si_7_O_9_(OH)_3_ [4]) are considered more interesting due to their ability to incorporate distinct functionalities by reaction with specific organosilanes or heteroelement precursors (i.e., metal halides or alkoxides) [5,6,7,8]. In particular, POSS have been combined with different metallic elements over the years, including alkali and alkaline-earth metals [9,10,11,12,13], metalloids [14,15,16,17], transition and post-transition metals [8,17,18], and lanthanides and actinides [11,19,20,21,22,23,24,25]. The preparation of metal-containing POSS (M-POSS) can be achieved through different strategies, such as (i) a corner-capping mechanism, in which the Si-OH groups of open-corner POSS react with the metals, forming fully condensed M-POSS [2,26,27]; (ii) a complexation reaction, in which organic ligand arms bound to the silicon vertices of the POSS core coordinate the metals [28,29,30]; (iii) interaction of anionic Si-O^−^ units of the POSS cage with metal ions, which is exploited to link several silsesquioxanes together via Si-O-M bridges [24].

When multiple POSS are connected together, they can act as structural nodes in the establishment of a polymeric-like network, which falls within the category of materials known as POSS-based polysilsesquioxanes (or simply PSQs) [31,32,33]. In addition, POSS units can be further modified with specific functionalities (i.e., magnetic, luminescent, or catalytic species) to design innovative hybrid 3D silica-based materials. POSS-based PSQs have been prepared in two distinct architectures: (i) hierarchical hybrid materials with high specific surface area and hydrothermal stability and variable micro-/meso-porosities, obtained from template-assisted condensation of POSS cages under acidic or basic pH [22,34,35,36,37]; (ii) amorphous or ladder-like PSQ structures, attained from long thermal treatments. New sustainable procedures have also been proposed to design amorphous PSQs, i.e., by self-condensation of open-corner T_7_-POSS [32].

Rare-earth metals, especially of the lanthanide series (*Ln*), represent critical elements for the scientific and technological development of humankind [38] due to their unique magnetic, electronic, optical, and catalytic properties [39,40,41,42,43]. Indeed, they are widely known for their sharp and well-defined absorption and emission bands in the UV-Vis-NIR spectral region, long-lived excited state lifetimes, large Stokes shift, and good photostability, as well as for variable electronic relaxation times and high effective magnetic moments [39,40]. Owning to these features, they have aroused great interest in many fields, i.e., for optical sensors, light-emitting diodes (LED), and in optical imaging and diagnostics [41,43,44,45,46]. The incorporation of luminescent lanthanides in molecular POSS and polymeric PSQ structures, for example, can be extremely beneficial to isolate these metal centers in complex matrices, in order to confer them high thermal, mechanical, and chemical stabilities and modular optoelectronic properties [47].

In recent years, the research on new rare-earth (RE)-doped silsesquioxanes has been increasing, attracting growing attention for their potential applications, although the number of articles is still relatively small. Most studies have focused on the design of molecular *RE*-POSS, i.e., with Ce^3+^ [48], Gd^3+^ [49], Dy^3+^ and Y^3+^ [50], Er^3+^ [51], Tb^3+^ and Eu^3+^ centers [19,52,53]. The incorporation of Eu^3+^ ions into a polysilsesquioxane network based on octa(tetramethylammonium)-POSS cages (OctaTMA POSS, a fully-closed T_8_-POSS) instead, led to a stable POSS-based mesoporous material with intrinsic optical properties [22]. Recently, our group proposed new luminescent and water-stable amorphous PSQs derived from the condensation of anionic OctaTMA POSS units and Eu^3+^ ions in simple and sustainable conditions, in which the metal acted as both a structural and functional agent [24]. Nonetheless, very few examples of silsesquioxanes simultaneously hosting different lanthanide ions have been reported so far [25,54,55]. Indeed, the interest towards co-doped lanthanide-based systems is continuously rising, since different properties can be inferred to the resulting materials by appropriate selection of the ion pairs [56], such as upconversion or tuneable magnetic and luminescent features [57].

Based on these considerations, in this study we describe the synthesis of novel co-doped luminescent amorphous PSQs with both Tb^3+^ and Eu^3+^ ions directly integrated in the POSS-based polysilsesquioxane matrix, following an optimized version of our previous synthetic protocol [24]. Two co-doped solid materials were prepared in water at room temperature and slightly acidic pH, followed by simple water evaporation, from the combination of completely condensed anionic OctaTMA POSS with lanthanide acetate precursors, using different Tb^3+^:Eu^3+^ molar ratios. The Si-O^−^ groups of the T_8_-POSS coordinate the Ln^3+^ ions, which act as metal bridges between the POSS cages. A mono-doped Tb-PSQ was also prepared as a reference solid. The effect of the different stoichiometric ratios of metal ions was then evaluated by multi-technique physico-chemical characterization to better understand the structure–property relationship of the final materials.

## 2. Materials and Methods

### 2.1. Materials

Octa(tetramethylammonium)-POSS (OctaTMA-POSS) was purchased form Hybrid Plastics (Hattiesburg, MS, USA) and used as received; hydrochloric acid (37%), xylenol orange and lanthanide acetate precursors were purchased form Sigma-Aldrich (Milano, Italy) and used as received unless otherwise specified.

### 2.2. Synthesis of Lanthanide-Doped PSQ

#### 2.2.1. Synthesis of Tb-PSQ

Tb-PSQ was prepared by condensation reaction of OctaTMA-POSS with terbium acetate, following the synthetic protocol adopted in the literature by S. Marchesi et al. for the preparation of mono-lanthanide doped polysilsesquioxanes through the combination of OctaTMA-POSS and lanthanide precursor [24]. The mol/mol ratio of POSS and terbium precursors was fixed at 1:1.

In detail, anhydrous terbium acetate (Tb(CH_3_COO)_3_; 151 mg, 0.451 mmol; Sigma Aldrich) was added with vigorous stirring to a solution of OctaTMA-POSS (POSS; 1 g, 0.451 mmol; Hybrid Plastics) in 20 mL of MilliQ^®^ water, previously adjusted to pH 5.5–5.6 with the addition of 1 M HCl solution. After addition of the reactants, the mixture was stirred at room temperature for 10 min and then transferred in an oven and heated at 80 °C until complete water evaporation (ca. 24 h). The powder was washed with pure water to remove unreacted compounds and by-products, obtaining the Tb-PSQ sample.

#### 2.2.2. Synthesis of Tb_(1−x)_Eu_(x)_-PSQ (TbEu-PSQA and TbEu-PSQB)

Co-doped Tb_(1−x)_Eu_(x)_-PSQ samples were prepared from a modified version of the synthesis mentioned before. Two samples with different stoichiometric ratios of lanthanide ions were prepared, one with x = 0.3 (Tb_0.7_Eu_0.3_-PSQ) named TbEu-PSQA, and one with x = 0.7 (Tb_0.3_Eu_0.7_-PSQ) called TbEu-PSQB. The mol/mol ratio of POSS and total metal precursors was fixed at 1:1.

In detail, a solid mixture of anhydrous terbium acetate (Tb(CH_3_COO)_3_; 106 mg, 0.315 mmol for TbEu-PSQA and 45 mg, 0.134 mmol for TbEu-PSQB) and europium acetate (Eu(CH_3_COO)_3_; 44 mg, 0.134 mmol for TbEu-PSQA and 104 mg, 0.316 mmol for TbEu-PSQB) was added to a solution of OctaTMA-POSS (POSS; 1 g, 0.451 mmol) in 20 mL of MilliQ^®^ water, previously adjusted to pH 5.5 by the addition of 1 M HCl solution. The solution was stirred for 10 min at room temperature (25 °C), and then it was transferred to an oven and heated at 80 °C until complete water evaporation. The final powder was washed with pure water in order to remove soluble reactants and by-products (POSS and lanthanide precursors).

### 2.3. Characterization

The washing of the three lanthanide-PSQs was carried out by redispersing the solid in water, followed by centrifugation (8000 rpm, 3 min); the supernatant was tested with the xylenol orange test in order to verify the presence of unreacted lanthanide precursors [58]. Briefly, an aliquot (200 μL) of supernatant was treated with 2 mL of xylenol orange solution, previously prepared by dissolving 3 mg of the chromophore in 250 mL of acetate buffer solution (50 mM, pH = 5.8) and characterized by UV-Visible spectroscopy. UV-Visible spectra in the 300–800 nm range were collected by using a Perkin Elmer Lambda 900 spectrometer (Waltham, MA, USA).

Energy dispersive X-ray (EDX) spectroscopy analyses were carried out on a FEG-SEM TESCAN S9000G instrument (Brno, Czech Republic) equipped with Oxford microanalysis detector Ultim Max and AZtec Software (version 6.0).

Fourier-transform infrared spectroscopy (FTIR) measurements were performed on a Nicolet 57,700 Spectrometer (Thermo Optics; Waltham, MA, USA), operating in the 4000–400 cm^−1^ range with a resolution of 4 cm^−1^. IR spectra of the solids mixed with potassium bromide (KBr) pellets (0.5 wt.%) were measured in the absorbance mode at room temperature.

X-ray powder diffractograms (XRPDs) were collected on unoriented ground powders on a Bruker D8 Advance Powder Diffractometer (Karlsruhe, Germany), operating in Bragg-Brentano geometry, with a Cu anode target equipped with a Ni filter (used as X-ray source) and with a Lynxeye XE-T high-resolution position-sensitive detector. Trio and Twin/Twin optics are mounted on the DaVinci.Design modular XRD system. The X-ray tube of the instrument operates with a Cu-K_α1_ monochromatic radiation (λ = 1.54062 Å), with the current intensity and operative electric potential difference set to 40 mA and 40 kV, respectively, and with automatic variable primary divergent slits and primary and secondary Soller slits of 2.5°. The X-ray profiles were recorded at room temperature in the 5°–60° 2θ range with a coupled 2θ−θ method, continuous PSD fast scan mode, time per step (rate or scan speed) of 0.100 s/step, and 2θ step size (or increment) of 0.01°, with automatic synchronization of the air scatter (or anti-scatter) knife and slits and with the fixed illumination sample set at 15 mm.

Dynamic light scattering (DLS) analysis was carried out in aqueous solution by using a Malvern Zetasizer NanoZS instrument (Malvern, UK) equipped with a He-Ne laser (λ = 633 nm). The DLS measures were performed at neutral pH and 25 °C. The sample suspensions were prepared by dispersing 1 mg of solid compound in 1 mL of ultrapure water and sonicating at 59 kHz for 5 min. Surface ζ-potential was quantified in water at 25 °C and neutral pH.

Photoluminescence (PL) spectra (excitation and emission) of the compounds in the solid state were recorded on a Horiba Jobin-Yvon Model IBH FL-322 Fluorolog 3 Spectrometer (Northampton, UK) equipped with a 450 W Xenon arc lamp, double grating excitation and emission monochromators (2.1 nm·mm^−1^ dispersion; 1200 grooves per mm) and a Hamamatsu Model R928 photomultiplier tube. Time-resolved measurements were carried out, instead, by using the time-correlated single-photon counting (TCSPC) option. A 370 nm spectraLED laser was employed to excite the samples at solid state and in H_2_O and D_2_O. The signal was collected using an IBH DataStation Hub photon counting module, while data analysis was accomplished with the commercial DAS6 software (version 6.6, HORIBA Jobin Yvon IBH).

## 3. Results and Discussion

Two co-doped samples defined as TbEu-PSQA and TbEu-PSQB were prepared by coordination of anionic OctaTMA-POSS in the presence of two diverse molar ratios of Eu^3+^ and Tb^3+^ precursors (TbEu-PSQA: Tb^3+^ = 0.7, Eu^3+^ = 0.3; TbEu-PSQB: Tb^3+^ = 0.3, Eu^3+^ = 0.7). A 1:1 molar ratio of POSS and total lanthanide precursors was applied in both preparations. The general synthetic procedure is an optimized co-doped variant of our previously published synthesis regarding the preparation of mono-lanthanide doped PSQs with structural POSS units and Eu^3+^ ions [24]. During the reaction, both Tb^3+^ and Eu^3+^ metal ions coordinate the Si-O^−^ groups of anionic POSS molecules in different positions, thus forming a polycondensed network of POSS cages connected by lanthanide centers. A Tb^3+^-containing PSQ solid, hereafter named Tb-PSQ, was also prepared as reference following the same experimental procedure. When necessary, a comparison was made of the data of the co-doped materials with those of the starting OctaTMA-POSS molecule and of the metal-free PSQ obtained in the previous study [24], in which the POSS cages co-condensed through simple siloxane bonds. The reaction was performed in a slightly acidic aqueous medium (pH 5.5) to avoid metal hydroxide precipitation [59], for 10 min at room temperature. It was subsequently followed by an aging step at 80 °C to allow slow water evaporation. The obtained solids were washed with water to remove any unreacted species (both POSS and lanthanide precursors); after each washing step, the amount of free metal ions present in the supernatant of each sample was examined with UV-Visible spectroscopy in the presence of a complexometric probe (xylenol orange). The chromophore shows a different color when it is in interaction with free metal ions, and it is one of the most common ‘indicators’ for free lanthanide ion detection adopted in the literature [58]. The UV-Vis spectrum of the free dye in acetate buffer at pH = 5.8 is defined by two separate absorptions at 432 and 578 nm, which are attributed to π → π* electronic transitions (Figure S1) [58]. The absorbance ratio of the two bands is sensitive to pH and to the presence of free lanthanide ions; thus, it is used to monitor the presence of free lanthanide ions in solution [58]. Tb-PSQ, TbEu-PSQA and TbEu-PSQB were washed until no perturbation of the intensity ratio of xylenol orange absorption bands was detected, confirming the absence of free Tb^3+^ and/or Eu^3+^ ions in the supernatant.

The actual loading of lanthanide dopants (in particular, the Tb^3+^ to Eu^3+^ ratio) was evaluated by EDX (Energy Dispersive X-ray) analysis (Figure S2), confirming that the desired stoichiometry was achieved, as reported in Table 1. In the Tb-PSQ reference sample, only Tb^3+^ was detected as the dopant, as expected.

**Figure 1:**
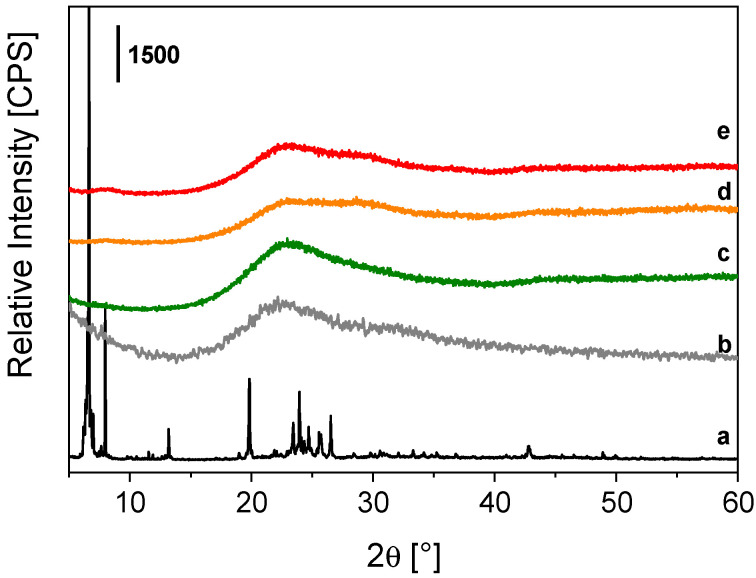
X-ray powder profiles of OctaTMA POSS (a), PSQ [24] (b), Tb-PSQ (c), TbEu-PSQA (d) and TbEu-PSQB (e).

The structural features of TbEu-PSQ samples were analyzed by the X-ray powder diffraction technique and compared to those of the reference Tb-PSQ solid, the metal-free PSQ material [24] and the OctaTMA-POSS reactant. The X-ray patterns of the co-doped solids (Figure 1, curves d and e) are similar, both being characterized by a broad signal in the 15–35° 2θ range, typically observed in amorphous silica materials and in the case of the reference PSQ [24] (Figure 1, curve b), with a seemingly multi-component nature. This last feature is more pronounced in TbEu-PSQA (Figure 1, d) and TbEu-PSQB (Figure 1, e) samples compared to Tb-PSQ (Figure 1, c). When comparing the XRD patterns of condensed samples (both PSQ and lanthanide-PSQs) to the one of OctaTMA-POSS, it is observed that the intense and well-resolved crystallographic reflections of OctaTMA-POSS (Figure 1, curve a) are completely lost after condensation of the anionic POSS cages. This indicates that the ordered organization of the silsesquioxane molecules is totally lost, suggesting an asymmetric and irregular re-organization of the POSS cages in polysilsesquioxane chains without the presence of ladder-like domains [60]. These observations are in agreement with what was already observed in the case of Eu-PSQ systems [24].

**Figure 2:**
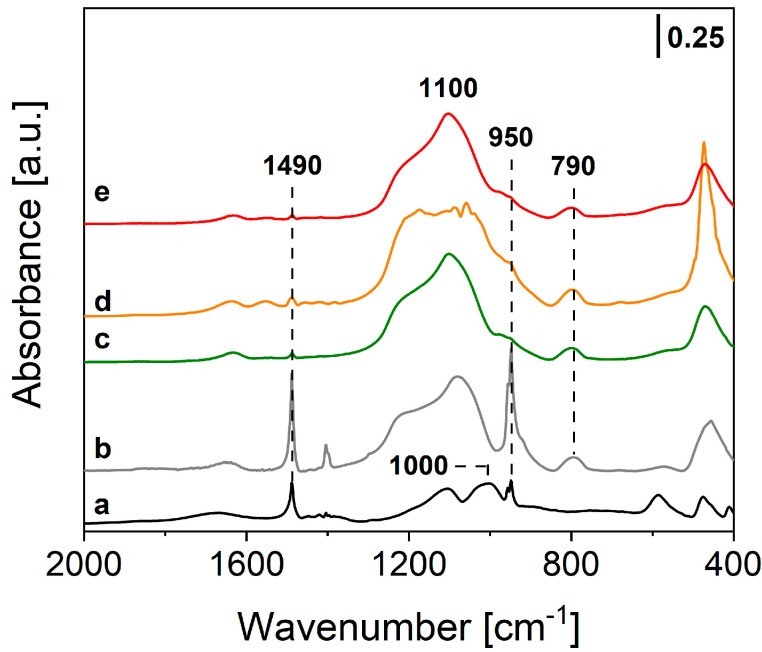
FTIR spectra of OctaTMA POSS (a), PSQ [24] (b), Tb-PSQ (c), TbEu-PSQA (d) and TbEu-PSQB (e), diluted in the KBr matrix (at 0.5 wt.%).

Further information on the local structure of the final samples was obtained through FTIR spectroscopy with the powders dispersed in KBr matrix (0.5 wt.%). FTIR spectra of Tb-PSQ, TbEu-PSQA and TbEu-PSQB are reported in Figure 2 and compared to the FTIR spectrum of OctaTMA-POSS. The FTIR spectrum of OctaTMA POSS (Figure 2, curve a) presents two main absorptions at ca. 1100 and 1000 cm^−1^ of the asymmetric stretching of Si-O-Si and Si-O^−^ groups of the POSS cage [61], and two narrow peaks at 1490 and 950 cm^−1^ due to bending modes of -CH_3_ and symmetric stretching of the C-N bond of tetramethyl ammonium (TMA) groups, which are in interaction with O^−^ atoms of the inorganic cage [62]. After complexation of the POSS units with Tb^3+^ (Figure 2, curve c) or both Tb^3+^ and Eu^3+^ ions in different stoichiometric ratios (Figure 2, curves d and e), a coalescence of the bands at 1000 and 1100 cm^−1^ is observed, leading to a wide multi-component absorption in the 1200–1000 cm^−1^ range of the spectrum. This feature can be ascribed to different inorganic architectures of the Si-O-Si groups of POSS cages after the polycondensation mechanism [24,32]. The same characteristics are found in the self-condensed reference PSQ material (Figure 2, curve b). Moreover, the signals of the TMA groups at 1490 and 950 cm^−1^ are almost completely eroded in the mono and co-doped samples, while some residuals are still visible in PSQ; this behavior is due to replacement of the organic groups in the presence of Tb^3+^ or both Tb^3+^/Eu^3+^ metal ions in the final polycondensed inorganic network, as previously observed in the literature for parent Eu-PSQ materials [24].

The aggregation state of co-doped TbEu-PSQA and TbEu-PSQB solids in water solution was analyzed by the dynamic light scattering technique. The suspensions of the materials were prepared by mixing 1 mg of each sample with 1 mL of pure water. DLS and surface ζ-potential analysis were carried out at neutral pH and 25 °C. The solid suspensions appear qualitatively homogeneous in the water medium. They are mainly composed by some aggregates of particles with distribution of hydrodynamic diameters centered at 220 and 345 nm for TbEu-PSQA and TbEu-PSQB, respectively, with a polydispersity index (PDI) of 0.74 and 0.51 (Figure S3). The high PDI value registered in both cases suggests the presence of particles with varying sizes, as testified by the broadness of DLS distributions. The DLS measurements were found to be highly reproducible, and the data meet the analysis quality criteria. Furthermore, both co-doped lanthanide-PSQs present a negative surface charge density due to the presence of several free Si-O^−^ groups after synthesis, as demonstrated by the values of surface ζ-potential reported in Table S1, thus corroborating the FTIR data.

**Figure 3:**
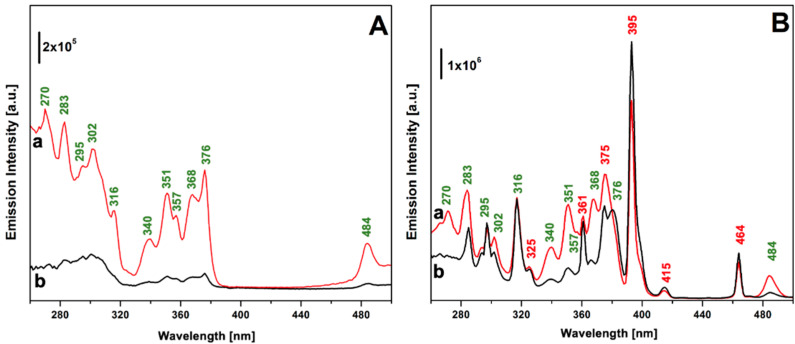
Excitation spectra of TbEu-PSQA (curve a) and TbEu-PSQB (curve b) monitored at 545 nm (section (**A**)) and 615 nm (section (**B**)). Wavelength associated with electronic transition assigned to Tb^3+^ and Eu^3+^ are labelled in green and in red, respectively.

The photophysical properties of TbEu-PSQ samples in the solid state were investigated by photoluminescence spectroscopy. Figure 3 shows the photoluminescence excitation spectra of the Tb^3+^/Eu^3+^-co-doped PSQs, monitored at 545 nm (section A) and at 615 nm (section B), corresponding to the most intense emission lines of Tb^3+^ and Eu^3+^, respectively. For the sake of comparison, the excitation spectrum of Tb-PSQ monitored at 545 nm is reported in Figure S4. The excitation spectra obtained by monitoring the 545 nm emission are defined by the characteristic peaks attributed to Tb^3+^ *f-f* transitions; in particular, peaks at 484, 376, 368, 357, 351, 340 and 316 nm were assigned to transition from the ^7^F_6_ ground state to the excited levels ^5^D_4_, ^5^G_6_ and ^5^D_3_, ^5^L_10_, ^5^L_9_–^5^D_2_ and ^5^G_5_, ^5^D_1_, and ^5^H_7_, respectively [25,63,64,65]. Transitions from ^7^F_6_ to 5d, ^5^I_8_, ^5^F_4_, ^5^F_5_ and ^5^H_4_ together give rise to the complex set of bands in the 260–300 nm range. When the emission of Eu^3+^ at 615 nm is monitored (Figure 3B), signals ascribed to the *f-f* transitions of Eu^3+^ appear, namely ^7^F_0_ → ^5^D_2_ (464 nm), ^7^F_0–1_ → ^5^D_3_ (415 nm), ^7^F_0_ → ^5^L_6_ (395 nm), ^7^F_0_ → ^5^L_7–8_, ^5^G_j_ (375 nm), ^7^F_0_ → ^5^L_9–10_, ^7^F_0–1_ → ^5^D_4_ (360 nm) and ^7^F_0_ → ^5^H_j_ (325 nm) [25,63,64,65,66]. Signals ascribed to Tb^3+^ transition are also present, and this is a first clear indication that Tb^3+^ → Eu^3+^ energy transfer occurs [25,67,68,69]. For the sake of clarity, the peak wavelengths assigned to the different transitions are marked in Figure 3 close to the corresponding peaks.

**Figure 4:**
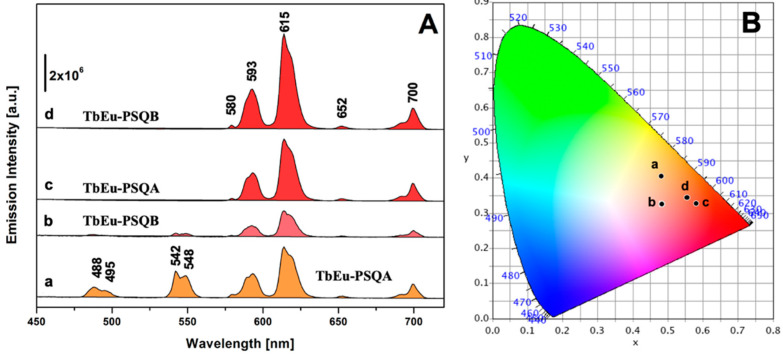
(**A**) Emission spectra of TbEu-PSQ solids excited at 270 nm (TbEu-PSQA, curve a; TbEu-PSQB, curve b) and at 395 nm (TbEu-PSQA, curve c; TbEu-PSQB, curve d). (**B**) CIE 1931 *xy* chromaticity diagrams derived from emission spectra in panel A. The *xy* coordinates of each spectrum are indicated in the diagram with a dot labelled with the same letter used in section (**A**). The color of each spectrum in section (**A**) corresponds to the *xy* coordinates identified in the CIE 1931 diagram.

Figure 4 reports the photoluminescence emission spectra of TbEu-PSQA and TbEu-PSQB under excitation at 270 nm, the λ_max_ of Tb^3+^ (Figure 4A, curves a and b) and at 395 nm, the λ_max_ of Eu^3+^ (Figure 4A, curves c and d). When excited at 395 nm, both the samples exhibit typical emission of Eu^3+^; in detail, the emission bands centered at 580, 593, 615, 652 and 700 nm are attributed to ^5^D_0_ → ^7^F_0_, ^5^D_0_ → ^7^F_1_, ^5^D_0_ → ^7^F_2_, ^5^D_0_ → ^7^F_3_ and ^5^D_0_ → ^7^F_4_ transitions [25,63,64,65,66], respectively, with the most intense line centered at 615 nm, as typical of Eu^3+^-doped materials [41,57]. Interestingly, upon excitation at 270 nm, where only Tb^3+^ is expected to be excited, in addition to the typical emission bands of Tb^3+^ in the 480–550 nm range (^5^D_4_ → ^7^F_6_ at 488–495 nm and ^5^D_4_ → ^7^F_5_ at 542–548 nm) both the co-doped samples exhibit the emission features of Eu^3+^ as previously discussed. This is a further strong confirmation of the metal-to-metal energy transfer occurring from Tb^3+^ to Eu^3+^ ions [25,67,68,69].

The photoemission stability of the co-doped systems was assessed by monitoring the photoemission intensity at 615 nm for 1 h, upon continuous illumination under 270 nm light. Both samples exhibited good photostability, as the photoemission intensity after 1 h of illumination was found to be 94% of the initial value in the case of TbEu-PSQA and 90% of the initial value in the case of TbEu-PSQB (Figure S5).

In order to gain insight into the effect of the Tb^3+^/Eu^3+^ ratio on the overall color of the co-doped sample emission, the photometric characteristics of TbEu-PSQA and TbEu-PSQB were derived from their emission spectra, calculating the *xy* chromaticity coordinates as well as related RGB, Hex and color purity parameters according to the CIE 1931 color space [70], as reported in Figure 4B and Table S2. Both samples, when excited at 395 nm, are characterized by red emission (dots c and d in Figure 4B), with high color purity. Excitation at 270 nm generates emissions with lower color purity and characterized by more orange-red color (dots a and b in Figure 4B).

As a first general insight into the local environment experienced by Eu^3+^ ions, the local geometry of Eu^3+^ sites can be estimated by the asymmetry factor (R_21_), defined as the ratio between the integrated intensity of ^5^D_0_–^7^F_2_ and ^5^D_0_–^7^F_1_ electronic transitions [71,72]. The asymmetry factor was calculated for TbEu-PSQA and TbEu-PSQB on the emission spectra obtained upon excitation at 395 nm (in order to have the contribution by Eu^3+^ emission only), considering the following spectral ranges for the integration: 602–638 nm (^5^D_0_–^7^F_2_ transition) and 581–602 nm (^5^D_0_–^7^F_1_ transition). The obtained R_21_ values were 2.13 for TbEu-PSQA and 2.36 in the case of TbEu-PSQB, indicating the heterogeneous nature of the coordination chemistry of Eu^3+^ in both co-doped materials.

The number of water molecules present in the first coordination sphere of the Tb^3+^ and Eu^3+^ centers (*q^Tb^* and *q^Eu^*, respectively) in TbEu-PSQA and TbEu-PSQB were determined by measuring the experimental lifetime values (*τ*) of the transitions at 545 (λ_em_ Tb) and 615 nm (λ_em_ Eu) (Table S3). The analyses were performed for the H_2_O and D_2_O dispersions of each solid under excitation at 370 nm with a SpectraLED source, taking advantage of the time-resolved fluorescence spectroscopy technique. The *q^Ln^* values were then derived from Equations (1) and (2) [73,74,75,76].

(1) qEu=1.20·(1τH2O−1τD2O−0.25)

(2) qTb=4.26·(1τH2O−1τD2O−0.06)

The photoluminescence decay curves of ^5^D_0_ and ^5^D_4_ excited states of Eu^3+^ and Tb^3+^ (Figure S6), respectively, were fitted with a double-exponential function, suggesting the presence of (at least) two types of different Tb^3+^/Eu^3+^ sites in the disordered polysilsesquioxane networks [77]. On average, from the above equations, a value of ca. 1–2 water molecules in coordination with Eu^3+^ or Tb^3+^ ions were found (Table 2); the low hydration state of both ions corroborates the previous structural data on the formation of a complex and unordered metal-containing polysilsesquioxane framework.

Lastly, the metal-to-metal energy transfer (MMET) process from Tb^3+^ to Eu^3+^ centers was investigated in more detail though measurements of *τ* values at the solid state of the donor Tb-PSQ (D) and donor–acceptor TbEu-PSQA and TbEu-PSQB (DA) systems (Table S3). The intensity decay curves of ^5^D_4_ excited state of the donor (or sensitizer) ion Tb^3+^ were collected at a λ_em_ of 545 nm under irradiation at 370 nm, in the presence (*τ_DA_*, Figure S7A) or absence (*τ_D_*, Figure S7B,C) of the acceptor (or activator) lanthanide (Eu^3+^). A bi-exponential fitting was applied to the experimental curves, extrapolating average *τ* values for each measurement. The energy transfer efficiency (*E_EnT_*) and rate (*k_EnT_*) parameters were then calculated from Equations (3) and (4) [67,68,78].

(3) $$\;k_{EnT}\;\left[s^{-1}\right]=\left(\frac{1}{\tau_{DA}}-\frac{1}{\tau_{D}}\right)$$

(4) $$E_{EnT}\;\left(o\;\eta_{sens}\right)\;\left[\%\right]=\left(1-\frac{\tau_{DA}}{\tau_{D}}\right)\cdot 100$$

The data analysis, reported in Table 2, showed an almost double energy transfer efficiency for the TbEu-PSQB sample, equal to ca. 60% versus a ca. 32% of the TbEu-PSQA solid. Moreover, the MMET rate appears to be faster for TbEu-PSQB, with a value equal to 1.24 × 10^3^ s^−1^ in comparison to 4.05 × 10^2^ s^−1^ for the TbEu-PSQA solid. This is in line with what is usually observed in energy transfer pairs, where to enhance the efficiency of the phenomenon the sensitizer concentration should be kept lower than the activator concentration [79]. The calculated parameters are comparable to other inorganic materials containing Tb^3+^ and Eu^3+^ ions in their structure as presented in the literature [67,69,78], such as the recent double-decker Tb^3+^/Eu^3+^-functionalized TetraSilanolPhenyl POSS materials proposed in Ref. [25].

## 4. Conclusions

In summary, novel luminescent amorphous PSQs co-doped with Tb^3+^ and Eu^3+^ ions directly integrated in the polysilsesquioxane matrix (Figure S8) were synthesized with a simple and sustainable approach in aqueous conditions and at room temperature, followed by water evaporation. Two co-doped materials were prepared by tuning the Tb^3+^/Eu^3+^ molar ratio, obtaining TbEu-PSQ solids characterized by high photostability and colors of the emitted light ranging from orange to deep red as a function of both the Tb^3+^/Eu^3+^ molar ratio and the chosen excitation wavelength. Despite the disordered nature of the obtained solids, confirmed by structural and spectroscopic characterization, a good energy transfer from Tb^3+^ to Eu^3+^ was observed, with higher efficiency displayed when sensitizer concentration was lower than activator concentration, as expected. Due to the easiness of preparation and their tunable photoluminescence emission properties, these materials are promising candidates for several applications, ranging from bioimaging to technological applications such as sensors, ratiometric luminescence-based thermometers and light-emitting components in inorganic or hybrid optical devices.

## Figures and Tables

**Table 1:** Tb^3+^/Eu^3+^ ratio in TbEu-PSQA and TbEu-PSQB solids obtained by EDX analyses.

Sample	Theoretical Tb/Eu Ratio	Measured Tb/Eu Ratio
TbEu-PSQA	2.33 ^1^	2.75
TbEu-PSQB	0.43 ^2^	0.45

^1^ Equal to a Tb^3+^:Eu^3+^ ratio of 0.7:0.3. ^2^ Equal to a Tb^3+^:Eu^3+^ ratio of 0.3:0.7.

**Table 2:** Hydration state of Tb^3+^ (*q^Tb^*) and Eu^3+^ (*q^Eu^*) ions, and Tb^3+^ → Eu^3+^ energy transfer rate (*k_EnT_*) and efficiency (*E_EnT_*) parameters of Tb-PSQ, TbEu-PSQA and TbEu-PSQB samples.

Sample	*q^Tb^*	*q^Eu^*	*k_EnT_* (s^−1^)	*E_EnT_* (%)
Tb-PSQ	1.61	-	-	-
TbEu-PSQA	-	1.48	4.05 × 10^2^	32.05
TbEu-PSQB	-	1.90	1.24 × 10^3^	59.16

## Data Availability

Not applicable.

## Supplementary Materials

The following supporting information can be downloaded at: https://www.mdpi.com/article/10.3390/ma15227996/s1, Figure S1: UV-Vis absorption spectra of Xylenol Orange (black curve) of the aqueous solution after washing procedure of TbEu-PSQA (red curve) and TbEu-PSQB (blue curve); Figure S2: FEG-SEM micrographs and corresponding EDX spectra of Tb-PSQ (**A**), TbEu-PSQA (**B**) and TbEu-PSQB (**C**) samples; Figure S3: Hydrodynamic diameters distribution in aqueous solution of TbEu-PSQA (a) and TbEu-PSQB (b); Figure S4: Excitation spectrum of Tb-PSQ, monitored at 545 nm.; Figure S5: Photostability of TbEu-PSQA (black squares) and TbEu-PSQB (red circles) solids under continuous excitation at 270 nm for 1 h; Figure S6: Normalized PL Tb^3+ 5^D_4_–^7^F_5_ (545 nm) intensity decay over time of Tb-PSQ in H_2_O (**A**) and D_2_O (**B**), under irradiation at 370 nm. Normalized PL Eu^3+ 5^D_0_–^7^F_2_ (615 nm) intensity decay over time of TbEu-PSQA in H_2_O (**C**) and D_2_O (**D**), under irradiation at 370 nm. Normalized PL Eu^3+ 5^D_0_–^7^F_2_ (615 nm) intensity decay over time of TbEu-PSQB in H_2_O (**E**) and D_2_O (**F**), under irradiation at 370 nm. The curves fitting was performed with a bi-exponential function (black lines). The *χ*^2^ and *RSS* (residual sum of squares) values are reported in the table below; Figure S7: Normalized PL Tb^3+ 5^D_4_–^7^F_5_ (545 nm) intensity decay over time of Tb-PSQ (**A**), TbEu-PSQA (**B**) and TbEu-PSQB (**C**), collected at the solid state under irradiation at 370 nm. The curves fitting was performed with a bi-exponential function (black lines). The *χ*^2^ and *RSS* (residual sum of squares) values are reported in the table below; Figure S8: Schematic representations of the Tb^3+^ and Eu^3+^-containing POSS-based Polysilsesquioxanes (TbEu-PSQ) samples; Table S1: Surface ζ-potential of TbEu-PSQA and TbEu-PSQB; Table S2: Photometric data, in accordance with CIE 1931 color space, of TbEu-PSQA and TbEu-PSQB excited at 270 nm and 395 nm (photoluminescence spectra reported in Figure 4A); Table S3: Experimental lifetimes (*τ* ) of Tb-PSQ (donor system, D) and TbEu-PSQA and TbEu-PSQB (donor-acceptor systems, DA), measured in H_2_O, D_2_O and at solid-state. In solution the τ values were collected by monitoring the main emissions of Tb^3+^ (λ_em_ 545 nm, ^5^D_4_–^7^F_5_ electronic transition) and Eu^3+^ (615 nm, ^5^D_0_–^7^F_2_), while at solid state they were analyzed at 545 nm. All the measurements were performed under irradiation at 370 nm.
