# Supplementary material for: Eu3+ and Tb3+ @ PSQ: Dual Luminescent Polyhedral Oligomeric Polysilsesquioxanes
Source: Materials (Basel). 2022 Nov 12;15(22):7996. doi: 10.3390/ma15227996 (PMC9694933; doi:10.3390/ma15227996)
Supplement: Supplementary file 1 [file materials-15-07996-s001.zip › materials-2004547-supplementary.pdf]

materials

Article

Eu^{3+} and Tb^{3+} @ PSQ: Dual Luminescent Polyhedral Oligomeric Polysilsesquioxanes

Stefano Marchesi [1,*], Ivana Miletto [2,*], Chiara Bisio [1,3], Enrica Gianotti [4], Leonardo Marchese [1] and Fabio Carniato [1]

1 Dipartimento di Scienze e Innovazione Tecnologica, Università del Piemonte Orientale, Viale Teresa Michel, 11, 15121 Alessandria, Italy
2 Dipartimento di Scienze del Farmaco, Università del Piemonte Orientale, Largo Donegani, 2/3, 28100 Novara, Italy
3 CNR-SCITEC Istituto di Scienze e Tecnologie Chimiche "Giulio Natta", Via G. Venezian, 21, 20133 Milano, Italy
4 Dipartimento per lo Sviluppo Sostenibile e la Transizione Ecologica, Università del Piemonte Orientale, Piazza Sant'Eusebio, 5, 13100 Vercelli, Italy
* Correspondence: stefano.marchesi@uniupo.it (S.M.); ivana.miletto@uniupo.it (I.M.); Tel.: +39-0131360216 (S.M.); +39-0321375747 (I.M.)

Abstract: The synthesis and characterization of novel luminescent amorphous POSS-based polysilsesquioxanes (PSQs) with Tb^{3+} and Eu^{3+} ions directly integrated in the polysilsesquioxane matrix is presented. Two different Tb^{3+}/Eu^{3+} molar ratios were applied, with the aim of disclosing the relationships between the nature and loading of the ions and the luminescence properties. Particular attention was given to the investigation of site geometry and hydration state of the metal centers in the inorganic framework, and of the effect of the $Tb^{3+} \rightarrow Eu^{3+}$ energy transfer on the overall optical properties of the co-doped materials. The obtained materials were characterized by high photostability and colors of the emitted light ranging from orange to deep red, as a function of both the Tb^{3+}/Eu^{3+} molar ratio and the chosen excitation wavelength. A good energy transfer was observed, with higher efficiency displayed when donor/sensitizer concentration is lower than acceptor/activator one. The easiness of preparation and the possibility to finely tune the photoluminescence properties make these materials valid candidates for several applications, ranging from bioimaging to sensors, ratiometric luminescence-based thermometers and optical components in inorganic or hybrid light-emitting devices.

Keywords: silsesquioxane; polysilsesquioxane; POSS; lanthanide; europium; terbium; coordination; luminescence; energy transfer; co-doped material

Citation: Marchesi, S.; Miletto, I.; Bisio, C.; Gianotti, E.; Marchese, L.; Carniato, F. Eu^{3+} and Tb^{3+} @ PSQ: Dual Luminescent Polyhedral Oligomeric Polysilsesquioxanes. *Materials* **2022**, *15*, 7996. https://doi.org/10.3390/ma15227996

Academic Editor: Adam Watras

Received: 17 October 2022
Accepted: 9 November 2022
Published: 12 November 2022

Publisher's Note: MDPI stays neutral with regard to jurisdictional claims in published maps and institutional affiliations.

Copyright: © 2022 by the authors. Licensee MDPI, Basel, Switzerland. This article is an open access article distributed under the terms and conditions of the Creative Commons Attribution (CC BY) license (https://creativecommons.org/licenses/by/4.0/).

1. Figures

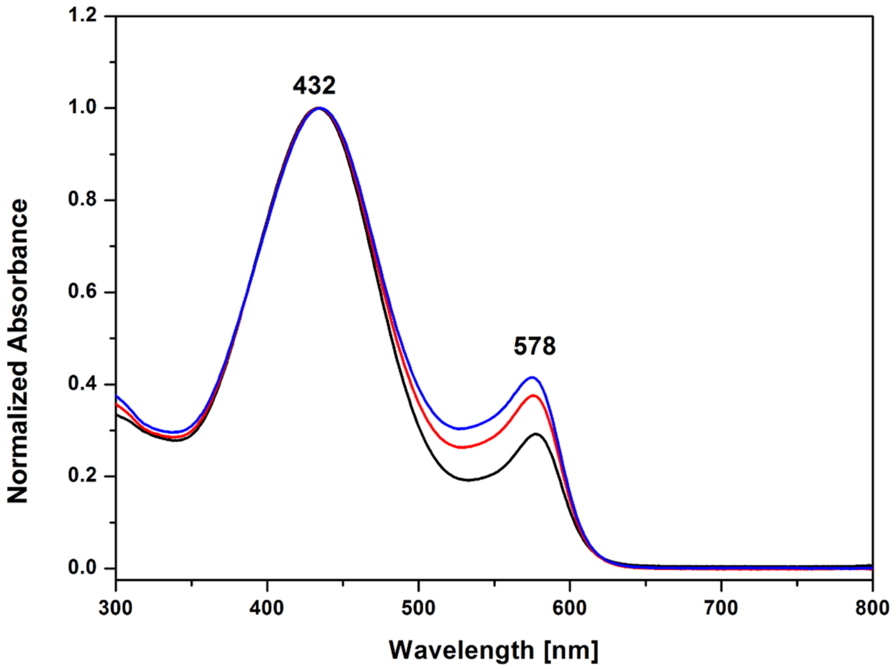

Figure S1. UV-Vis absorption spectra of Xylenol Orange (black curve) of the aqueous solution after washing procedure of TbEu-PSQA (red curve) and TbEu-PSQB (blue curve).

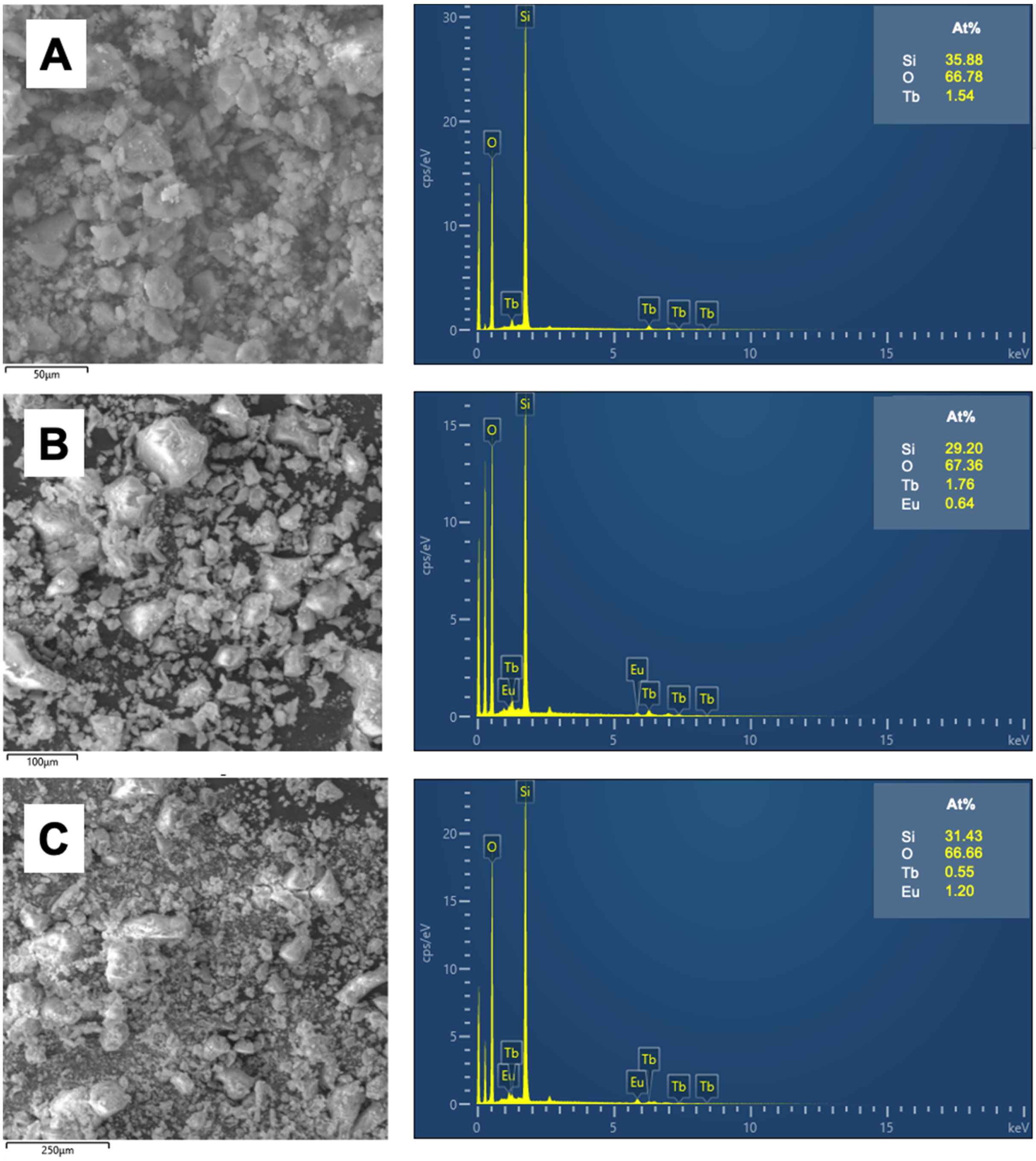

Figure S2. FEG-SEM micrographs and corresponding EDX spectra of Tb-PSQ (**A**), TbEu-PSQA (**B**) and TbEu-PSQB (**C**) samples.

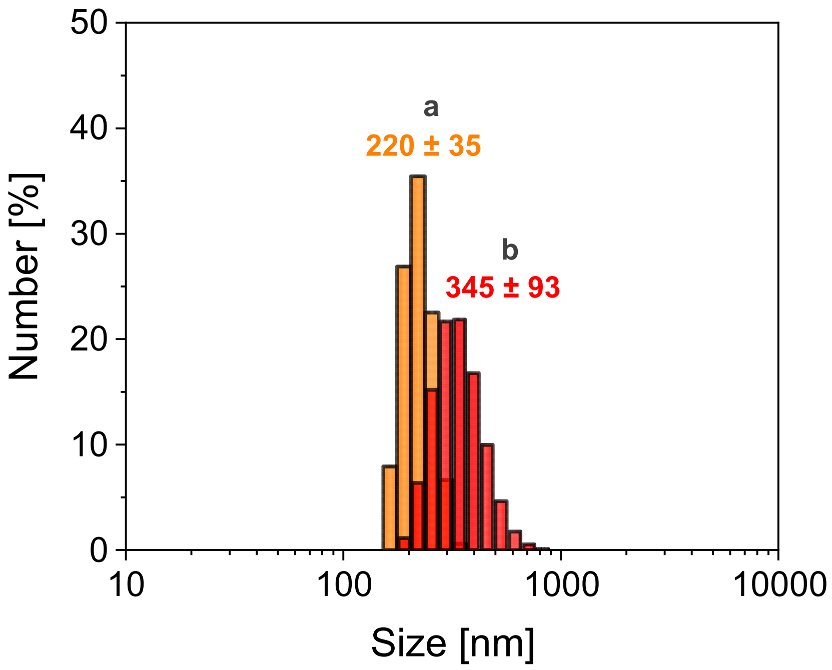

Figure S3. Hydrodynamic diameters distribution in aqueous solution of TbEu-PSQA (a) and TbEu-PSQB (b).

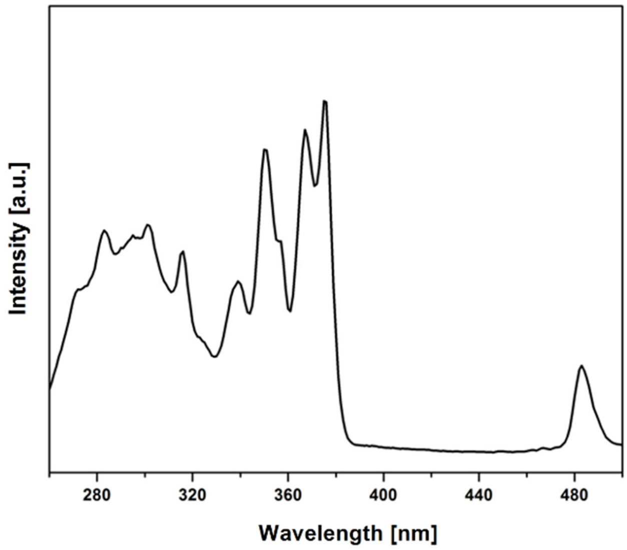

Figure S4. Excitation spectrum of Tb-PSQ monitored at 545 nm.

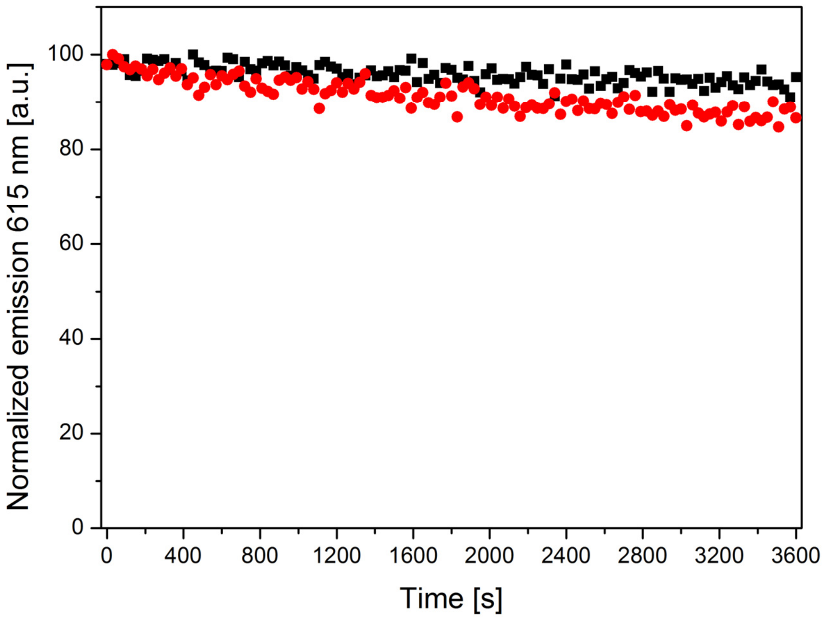

Figure S5. Photostability of TbEu-PSQA (black squares) and TbEu-PSQB (red circles) solids under continuous excitation at 270 nm for 1 h.

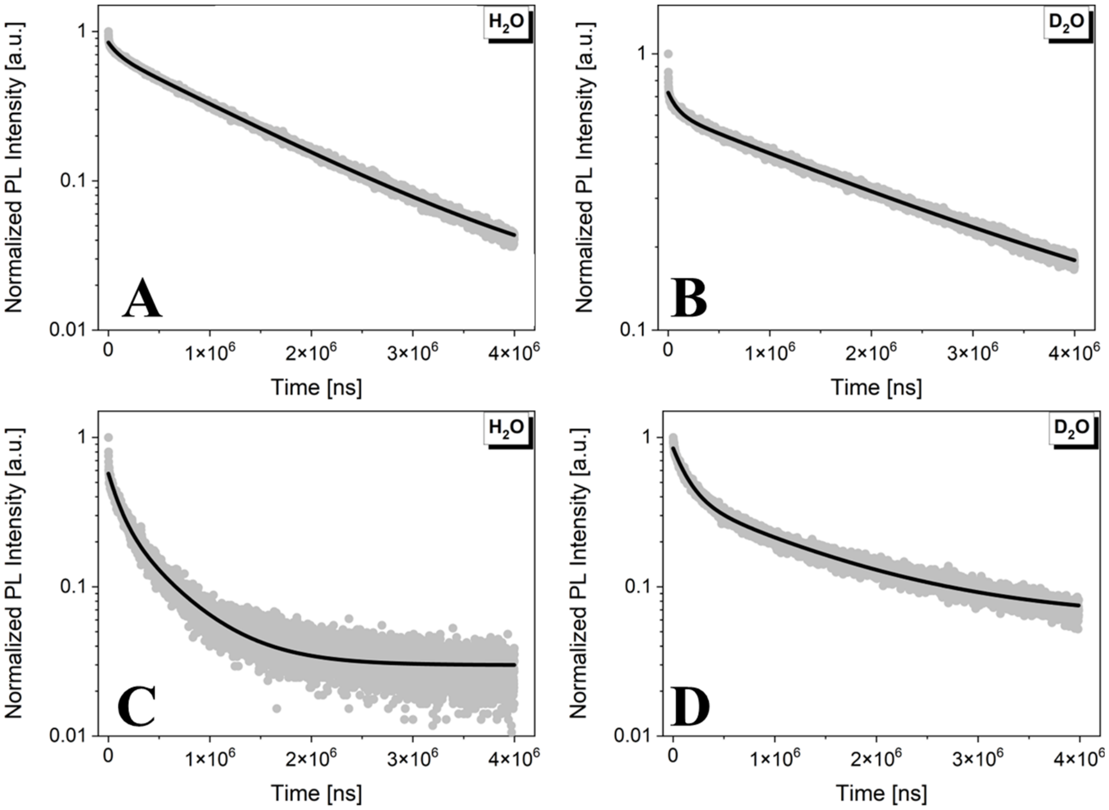

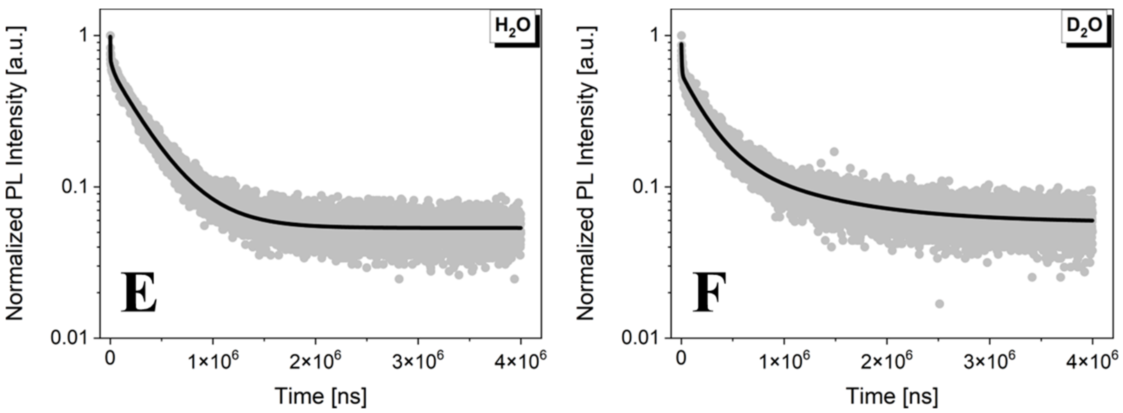

Figure S6. Normalized PL Tb^{3+} $^5D_4$-$^7F_5$ (545 nm) intensity decay over time of Tb-PSQ in H_2O (**A**) and D_2O (**B**), under irradiation at 370 nm. Normalized PL Eu^{3+} $^5D_0$-$^7F_2$ (615 nm) intensity decay over time of TbEu-PSQA in H_2O (**C**) and D_2O (**D**), under irradiation at 370 nm. Normalized PL Eu^{3+} $^5D_0$-$^7F_2$ (615 nm) intensity decay over time of TbEu-PSQB in H_2O (**E**) and D_2O (**F**), under irradiation at 370 nm. The curves fitting was performed with a bi-exponential function (black lines). The χ^2 and *RSS* (residual sum of squares) values are reported in the table below:.

**Sample**	χ^2	*RSS*
Tb-PSQ (H_2O)	4.049E-5	0.323
Tb-PSQ (D_2O)	6.215E-5	0.495
TbEu-PSQA (H_2O)	2.181E-4	1.744
TbEu-PSQA (D_2O)	1.317E-4	1.047
TbEu-PSQB (H_2O)	5.077E-4	4.053
TbEu-PSQB (D_2O)	2.600E-4	2.078

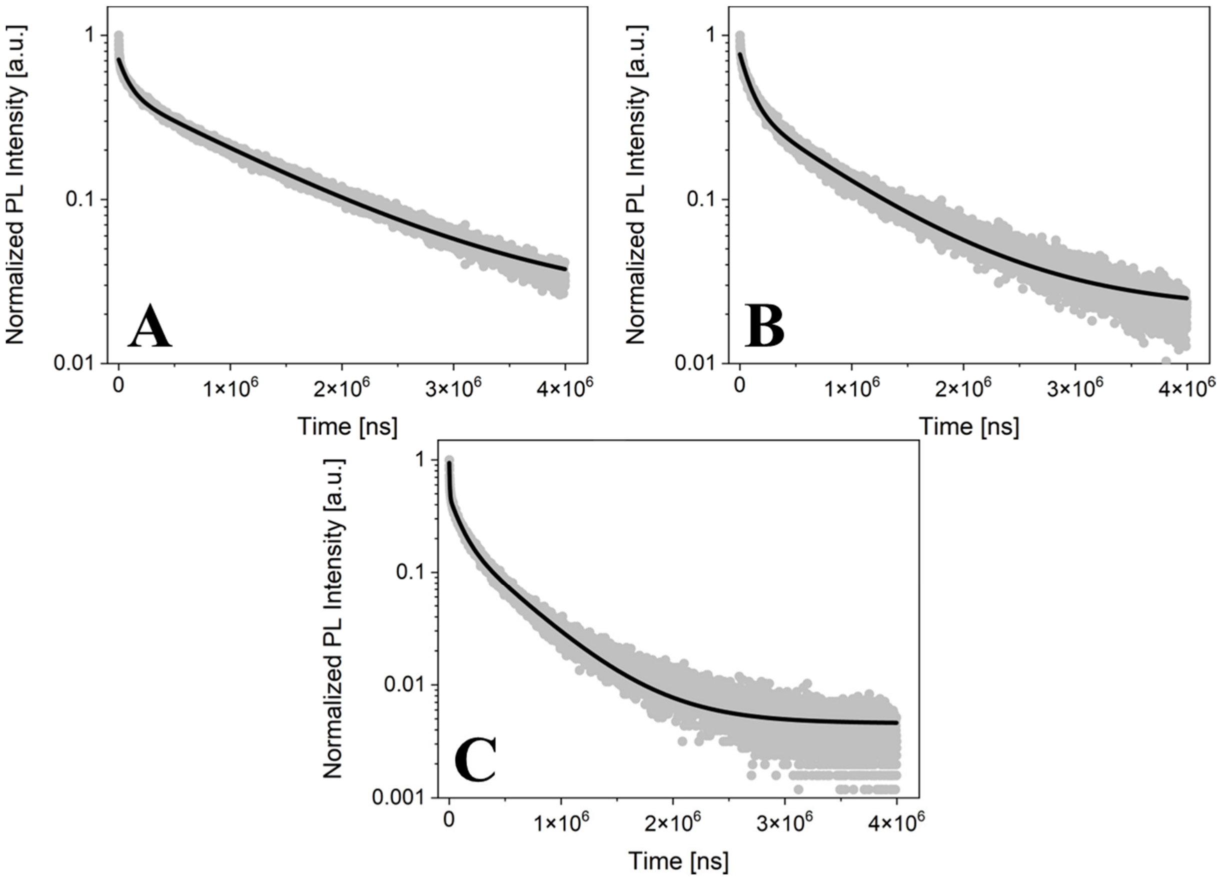

Figure S7. Normalized PL Tb^{3+} $^5D_4$-$^7F_5$ (545 nm) intensity decay over time of Tb-PSQ (**A**), TbEu-PSQA (**B**) and TbEu-PSQB (**C**), collected at the solid-state under irradiation at 370 nm. The curves fitting was performed with a bi-exponential function (black lines). The χ^2 and *RSS* (residual sum of squares) values are reported in the table below:.

**Sample**	χ^2	***RSS***
Tb-PSQ (D)	8.241E-4	0.657
TbEu-PSQA (DA)	1.020E-3	8.103
TbEu-PSQB (DA)	5.077E-4	4.053

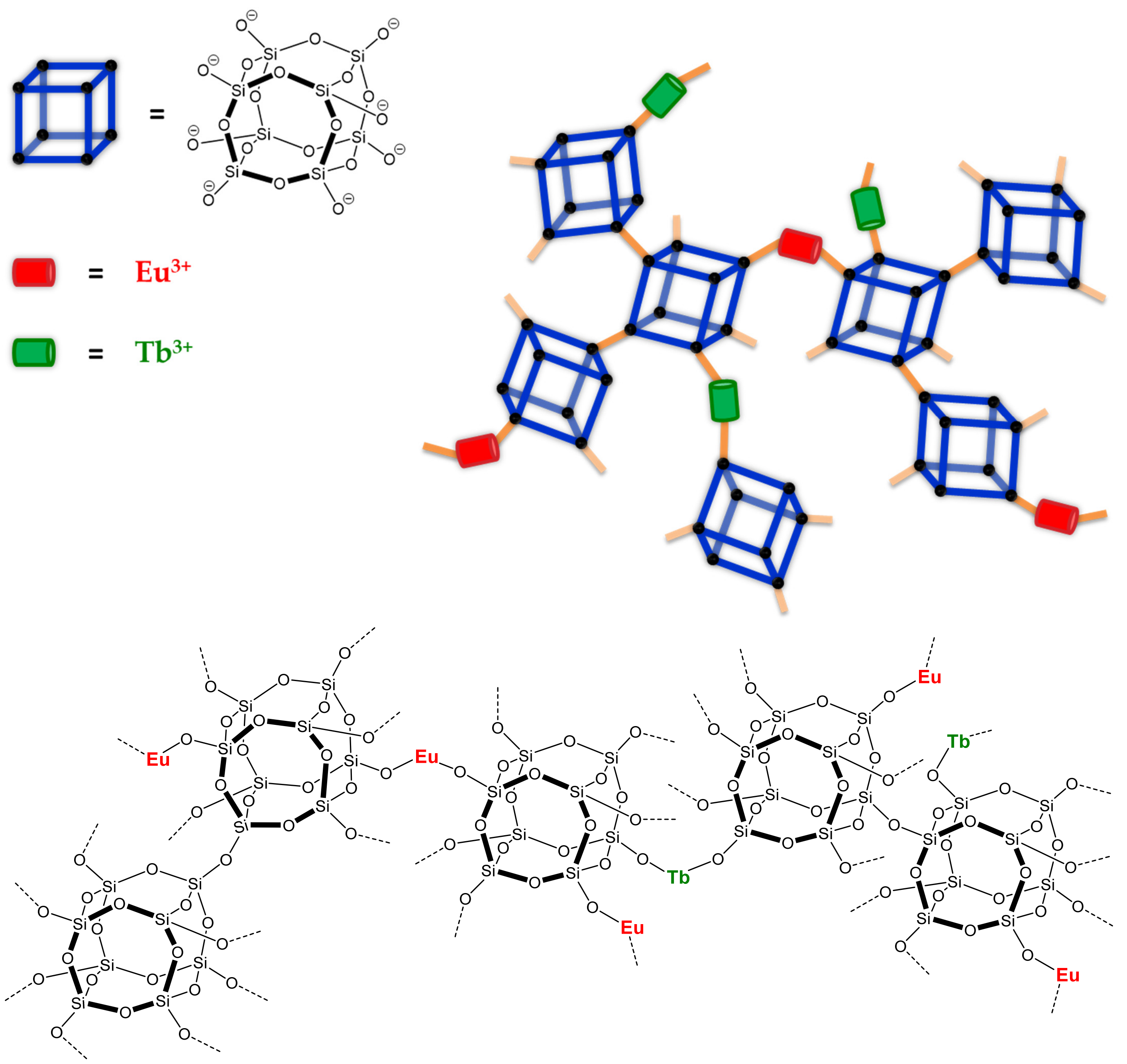

Figure S8. Schematic representations of the Tb^{3+} and Eu^{3+}-containing POSS-based Polysilsesquioxanes (TbEu-PSQ) samples.

2. Tables

Table S1. Surface ζ-potential of TbEu-PSQA and TbEu-PSQB.

Sample	Surface ζ-potential [mV]
TbEu-PSQA	-27.5 ± 7.7
TbEu-PSQB	-31.1 ± 4.9

Table S2. Photometric data, in accordance with CIE 1931 color space, of TbEu-PSQA and TbEu-PSQB excited at 270 nm and 395 nm (photoluminescence spectra reported in Figure 4A).

Sample	λ_{exc} [nm]	*x*	*y*	R	G	B	Hex	Color	Color Purity (%)
TbEu-PSQA	270	0.4848	0.4042	255	153	75	FF994B		66.8
TbEu-PSQB	270	0.4834	0.3272	255	110	112	FF6E70		43.2
TbEu-PSQA	395	0.5804	0.3276	255	60	63	FF3C3F		72.4
TbEu-PSQB	395	0.5594	0.3328	255	54	49	FF3631		79.7

Table S3. Experimental lifetimes (τ) of Tb-PSQ (donor system, D) and TbEu-PSQA and TbEu-PSQB (donor-acceptor systems, DA), measured in H_2O, D_2O and at solid-state. In solution the τ values were collected by monitoring the main emissions of Tb^{3+} (λ_{em} 545 nm, $^5D_4$-$^7F_5$ electronic transition) and Eu^{3+} (615 nm, $^5D_0$-$^7F_2$), while at solid-state they were analysed at 545 nm. All the measurements were performed under irradiation at 370 nm.

Sample	τH_2O [ms]	τD_2O [ms]	τ_D [ms]	τ_{DA} [ms]
Tb-PSQ	1.235	2.697	1.164	-
TbEu-PSQA	0.420	1.112	-	0.791
TbEu-PSQB	0.342	0.916	-	0.475
